# Single nucleotide polymorphisms of growth hormone gene in cattle and their association with growth traits: a systematic review

**DOI:** 10.1007/s11250-024-03985-1

**Published:** 2024-04-25

**Authors:** Lubabalo Bila, Dikeledi Petunia Malatji, Thobela Louis Tyasi

**Affiliations:** 1 Department of Animal Production, Potchefstroom College of Agriculture, Private Bag X1292, Potchefstroom, 2520 South Africa; 2 Department of Agriculture and Animal Health, College of Agriculture and Environmental Sciences, University of South Africa, Florida, 1710 South Africa; 3 Department of Agricultural Economics and Animal Production, School of Agricultural and Environmental Sciences, University of Limpopo, Private Bag X1106, Sovenga, Limpopo, 0727 South Africa

**Keywords:** Cattle, Growth traits, Growth hormone gene, Body weight, Weaning weight

## Abstract

Growth traits in livestock animals are quantitative parameters, which are often controlled by many genes including growth hormone (GH) gene. However, the evidence of effect of GH gene on growth traits of cattle is poorly understood. Hence, the objective of the study was to systematically investigate the literature on single nucleotide polymorphisms (SNPs) of GH gene and their association with growth traits in cattle from four databases Google Scholar, PubMed, ScienceDirect, and Web of Science. The results indicated that fifteen (*n* = 15) articles with 27% of them from Indonesia qualified to be used in this study after screening. The results revealed five SNPs (1047T > C, 1180 C > T, 86,273,136 A/G, 3338 A > G and 4251 C > T) occurred across multiple investigated breeds with no common identified SNPs. Six articles observed a significant difference (*p* < 0.05) between growth traits and genotypes of identified SNPs. The findings showed that 7 articles (47%) investigated body weight (BW) with 6 (40%) of them found non-significant and 1 (7%) found a significant association with genotypes of the identified SNPs (3338 A > G). While 7 articles (47%) investigated weaning weight (WW) with 5 (33%) of them revealed a non-significant and 2 (13%) found a significant association with genotypes of identified SNPs (3338 A > G and 4251 C > T). This study shows that there is a lack of evidence on effect of growth hormone gene on growth traits in cattle. However, more studies are recommended for further validation of the identified SNPs and effect of growth hormone gene on growth traits in cattle.

## Introduction

Growth Hormone (GH) is a protein hormone that is manufactured and secreted by the part of the pituitary gland (Agung et al. 2017). The hormone encoded by the GH gene and affects the productivity in beef cattle (Agung et al. 2017). Furthermore, the GH is necessary for tissue growth, fat metabolism and normal body growth (Agung et al. 2017). Agung et al. (2017) reported that the function of the GH is to play a role in reproduction traits such superovulation response, ovulation rate, fertility rate and embryo quality. The significance of the function of GH gene is to make this gene as one of candidate genes for marker assisted selection (MAS) program. Nowadays, molecular technology based on MAS has been widely used in cattle genetic improvement (Fathoni et al. 2019). Single nucleotide polymorphisms (SNPs) of GH gene and their association with growth traits in cattle has been investigated in different cattle breeds including Sumba Ongole (Agung et al. 2017), Holstein heifers (Arango et al. 2014), Colombian Creote cattle (Martinez et al. 2016), Pasundan cows (Putra et al., 2019) and Brangus bull (Thomas et al. 2007). However, based on authors’ knowledge there is no literature that has systematically investigated the SNPs of GH gene and their relationship with growth traits in cattle. To close the identified knowledge gap, the objective of the study was to systematically investigate the literature on single nucleotide polymorphisms of GH gene and their association with growth traits in cattle. This systematic review might help researchers and cattle farmers to know the genetic markers of GH gene that might be used for selection during breeding to improve growth traits in cattle.

## Materials and methods

### Eligibility criteria

Identification of Population, Exposure, and Outcomes (PEO) components of the research question as explained by Bettany-Saltikov (2010) was performed prior conducting the systematic review. Thereby the population was defined as “Cattle”, with an exposure of “Polymorphisms” and outcomes of “Growth Traits”. A pilot search of the PEO components on the Google Scholar database was conducted prior deciding to conduct the study.

### Literature search

The literature search for the research publications was done by all the authors through the use of four database Google Scholar, PubMed, ScienceDirect, and Web of Science up to 04 December, 2023 where the following keywords were made use of: ‘growth hormone gene (GH)’, ‘single nucleotide polymorphisms/genetic effect/polymorphisms/genetic variations’, ‘growth traits’ and ‘cattle’.

### Inclusion criteria

Titles and abstracts found using the search strategy were manually screened to recognize articles that were potentially appropriate. Articles were considered for inclusion in the systematic review given: some they included associations between growth hormone gene and growth traits in cattle.

### Exclusion criteria

Articles published in any other language other than English, only have abstracts accessible, only have English abstract, articles talking about different species other than cattle were excluded. Lastly, duplicate articles from the different database used were also excluded.

### Data extraction

The content was extracted independently by the authors. The contents extracted from the articles include the name of the 1st author, year the article was published, country, cattle breed, population size, investigated trait and genotyping method.

### Ethical considerations

Ethical consideration including plagiarism, misconduct, informed consent, data falsification, and fabrication were considered by all the authors.

## Results

### Literature searched results

**Fig. 1:**
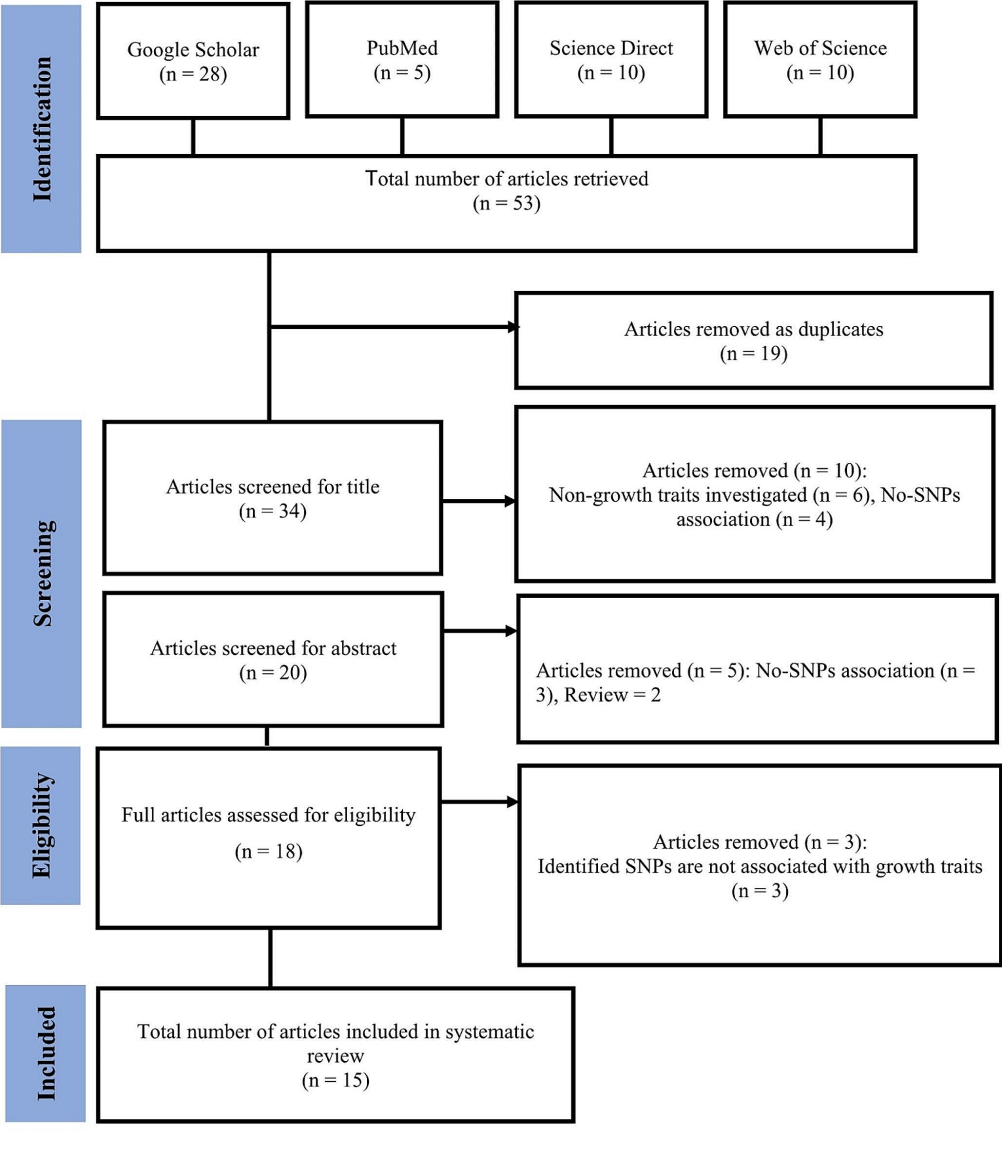
Flowchart of identification and selection of studies for systematic review

As indicated in Fig. 1, the articles retrieved for this systematic review amounted to a total of fifty-three (*n* = 53) from the following databases: Google Scholar (*n* = 28), PubMed (*n* = 5), Science Direct (*n* = 10), and Web of Science (*n* = 10). The duplicates (*n* = 19) that occurred in between the search databases were removed and the remaining articles were further analysed for inclusion and exclusion criteria. Finally, thirty four articles (*n* = 34) were screened on the basis of title, followed by the screening for the abstract, thereby eighteen (*n* = 18) articles were assessed for eligibility. A total of fifteen (*n* = 15) articles was used in writing this systematic review.

### Characteristics of included articles

About fifteen (*n* = 15) included articles of the fifty-three (*n* = 53) analysed were retained for inclusion in the literature review (Table 1). The retained articles ranged from the year 2003 to 2020. All included articles investigated growth hormone gene and its association with growth traits in cattle. The Angus cattle breed being most investigated (Ge et al. 2003; Kayumov et al. 2019; Ruban et al. unknown) accounting for 20% (*n* = 3) of the included articles.

**Table 1:** Characterization of included articles

Author	Year	Country	Breed	N	Trait	Genotyping method
Agung et al.	2017	Indonesia	Suma Ongole	267	BW, WW, YW	PCR-RFLP
Arango et al.	2014	Colombia	Holstein	480	W_E_, W_C_, DWG	PCR-RFLP
Beauchemin et al.	2006	United State	Brahman	324	ADG, FT, HCW, MBS, WG, RY, KPH	PCR- RFLP
China et al.	2007	Thailand	Thai Multibreed (Charolais, Brahman, Thai local native)	130	BW, WW	PCR- SSCP
Fathoni et al.	2019	Indonesia	Kebumen Ongole Grade cattle	100	BW_B_, BSH, BBL, BCC, WW, WSH, WBL, WCC, ADG, YW, YSH, YBL, WCC	PCR-RFLP
Garrett et al.	2008	Mexico	Brangus	553	BW, BW_205d_, BW_365d_, ADG_205d_, TADG, SC	PCR-RFLP
Ge et al.	2003	United State	Angus	-	BW, WW, OTW	PCR-RFLP
Kayumov et al.	2019	Russia (Kalmykia)	crossbred Red Angus × Kalmyk heifers	-	BW, WH, BL, CD	PCR-RFLP
Martinez et al.	2016	Colombia	Colombian creole cattle (ROMO, BON and Zebu)	386	WW, W_16M_	PCR-RFLP
Putra et al.	2019	Indonesia	Pasundan Cows	142	BL, WH, HG, BW	PCR-RFLP
Maskur & Arman	2014	Indonesia	Bali Cattle	250	ADG, BW, WW	PCR-RFLP.
Ruban et al.	Unknown	Ukraine	Angus cows	52	BW, W_8m_, W_12m_, W_15m_, W_18m_, W_2y_	PCR-RFLP
Sedykh et al.	2020	Russia	Hereford, Limousine, Black-and-White, Bestuzhev	629	ADG, LBW_B_, RGR	PCR-RFLP
Thomas et al.	2007	Mexico	Brangus bulls	434	BW, ADG, SC, BF	PCR-RFLP
Zhang et al.	2011	China	Chinese native cattle (Qinchuan and Nanyang)	240	BW_6m_, BW_12m_, BW_18m_, BW_24m_	PCR-SSCP

BW - Body weight, WW - Weaning weight, YW - Yearling weight, PCR - polymerase chain reaction, W_E_- Weight at first oestrus, W_C_ - Weight at first calving, DWG - Daily weight gain, ADG - average daily gain, FT - Fat thickness, HCW - Hot carcass weight, MBS - Marbling score, YG - Yield gain, RY - Retail yield, KPH fat - kidney, pelvic, heart, SSCP - single strand conformation polymorphism, BW_B_ - Body weight at birth, BSH - Birth Shoulder Height, BBL - Birth Body Length, BCC - Birth Chest Circumference, WSH - Weaning Shoulder Height, WBL - Weaning Body Length, WCC - Weaning Chest Circumference, YSH - Yearling Shoulder Height, YBL - Yearling Body Length, WCC - Yearling Chest Circumference, BW_205d_ − 205-days birth weight, BW_365d_ − 365-days birth weight, ADG_205d_ - Average daily gain at 205, TADG - Test Average daily gain, SC - Scrotal circumference, OTW, On test body weight, BL - body length, CD - chest depth, W16_M_ - Weight at 16 months, WH - withers height, HG - heart girth, W_8m_ - Weight at 8 months, W_12m_ - Weight at 12 months, W_15m_ - Weight at 15 months, W_18m_ - Weight at 18 months, W_2y_ - Weight at 2 years, LBW_B_ - Live body weight at birth, RGR - Relative growth rate, BF - back fat, BW_6m_ - body weight at 6 months, BW_24m_ - body weight at 24 months, RFLP - restriction fragment length polymorphism

### Origin of the publications

**Fig. 2:**
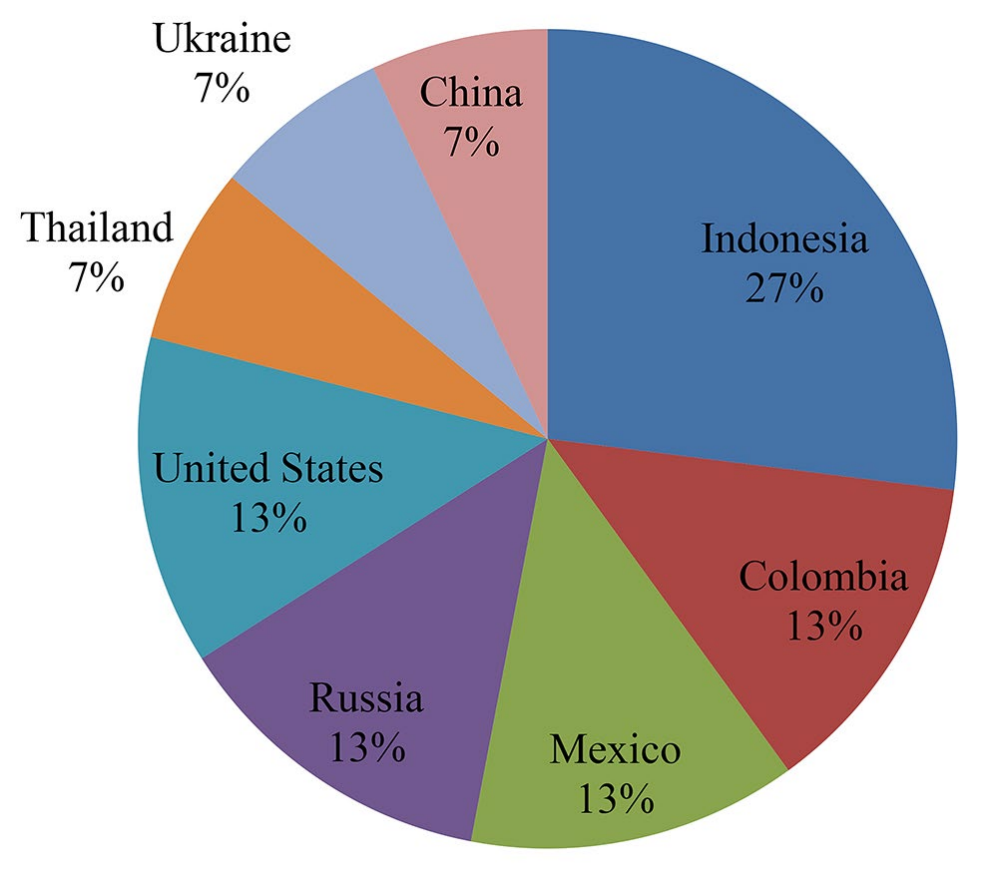
Publications by country

Article distribution per country is displayed in Fig. 2. All the included articles showed the origin of the publications. The results indicated that the included articles were from different countries such as Indonesia, Colombia, Mexico, Russia, United State, Thailand, Ukraine, and China. In addition, the findings revealed that the majority (27%) of the included articles were from Indonesia (Agung et al. 2017; Fathoni et al. 2019; Putra et al. 2019; Maskur & Arman 2014) followed by (13%) Colombia (Arango et al. 2014; Martinez et al. 2016), Mexico (Garrett et al. 2008; Thomas et al. 2007), Russia (Kayumov et al. 2019; Sedykh et al. 2020) and United State (Beauchemin et al. 2006; Ge et al. 2003), while the least (7%) included articles were from Thailand (China et al. 2007), Ukraine (Ruban et al. unknown), and China (Zhang et al. 2011).

### Distribution of the articles by known and unknown years

**Fig. 3:**
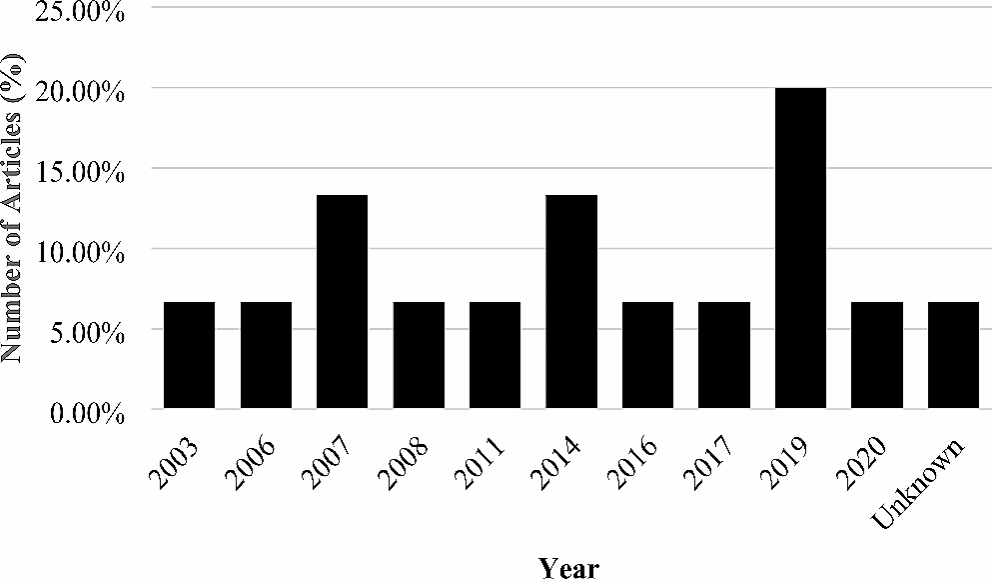
Distribution of articles by year

Article distribution per year is presented in Fig. 3. The results revealed that fourteen articles indicated the year of publication while only one article did not reveal the year of publication. Moreover, the findings of the review indicated that included articles were published in the year 2003 (6.66%), 2006 (6.66%), 2007 (13.33%), 2008 (6.66%), 2011 (6.66%), 2014 (13.33%), 2016 (6.66%), 2017 (6.66%), 2019 (20%), 2020 (6.66%) and unknown (6.66%). The results showed that most included articles were published in year 2019 (Fathoni et al. 2019; Putra et al. 2019; Kayumov et al. 2019).

### Publications by journals

Article distribution per journals is displayed in Table 2. The findings indicated that included articles were published in different journals such as Journal of the Indonesian Tropical Animal Agriculture, Genetics and Molecular Research Online Journal, Kasetsart Journal of Natural Science, Journal of Animal Science, Advances in Intelligent Systems Research, Tropical Animal Science Journal, Italian Journal of Animal Science, unknown, Iranian Journal of Applied Animal Science and Research in Veterinary Science. The results showed that most of the included articles were published by the Genetics and Molecular Research Online Journal with 27% (Arango et al. 2014; Beauchemin et al. 2006; Martinez et al. 2016; Thomas et al. 2007).

**Table 2:** Publications by journals

Journal	Number of articles
Genetics and Molecular Research Online Journal	4
Journal of the Indonesian Tropical Animal Agriculture	2
Advances in Intelligent Systems Research	1
Tropical Animal Science Journal	1
Italian Journal of Animal Science	1
None	1
Iranian Journal of Applied Animal Science	1
Research in Veterinary Science	1

### Distribution of the articles by genotyping methods

**Fig. 4:**
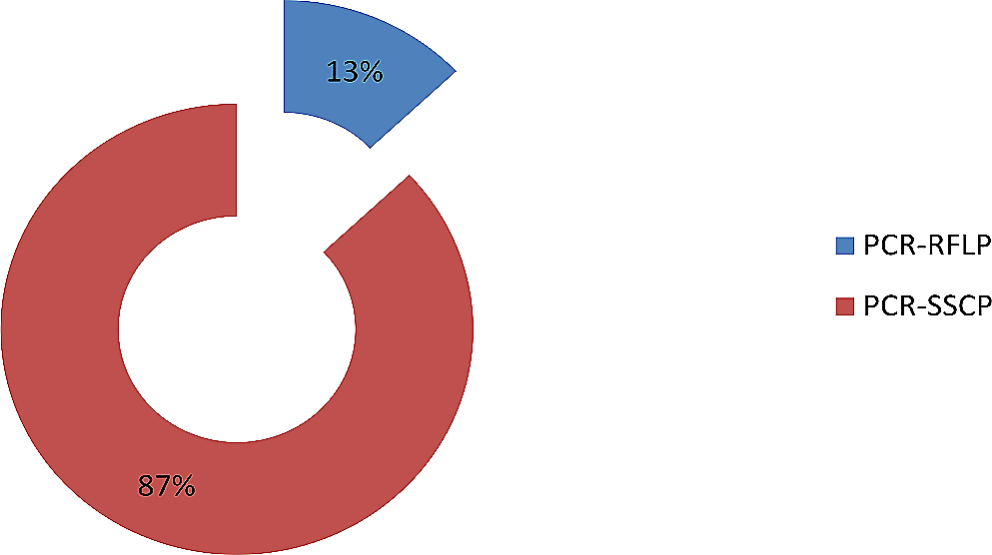
Distribution of the articles by genotyping method

Article distributions by genotyping methods are presented in Fig. 4. The results indicated that Polymerase Chain Reaction-Restriction Fragment Length Polymorphism (PCR-RFLP) and Polymerase Chain Reaction-Single Strand Conformation Polymorphism (PCR-SSCP) were the genotyping methods used by the included articles. The results showed that most included articles 87% (*n* = 13) used the PCR-RFLP method for genotyping while 13% (*n* = 2) of the included articles (China et al. 2007; Zhang et al. 2011) used PCR-SSCP genotyping method.

### Articles by genomic regions

**Fig. 5:**
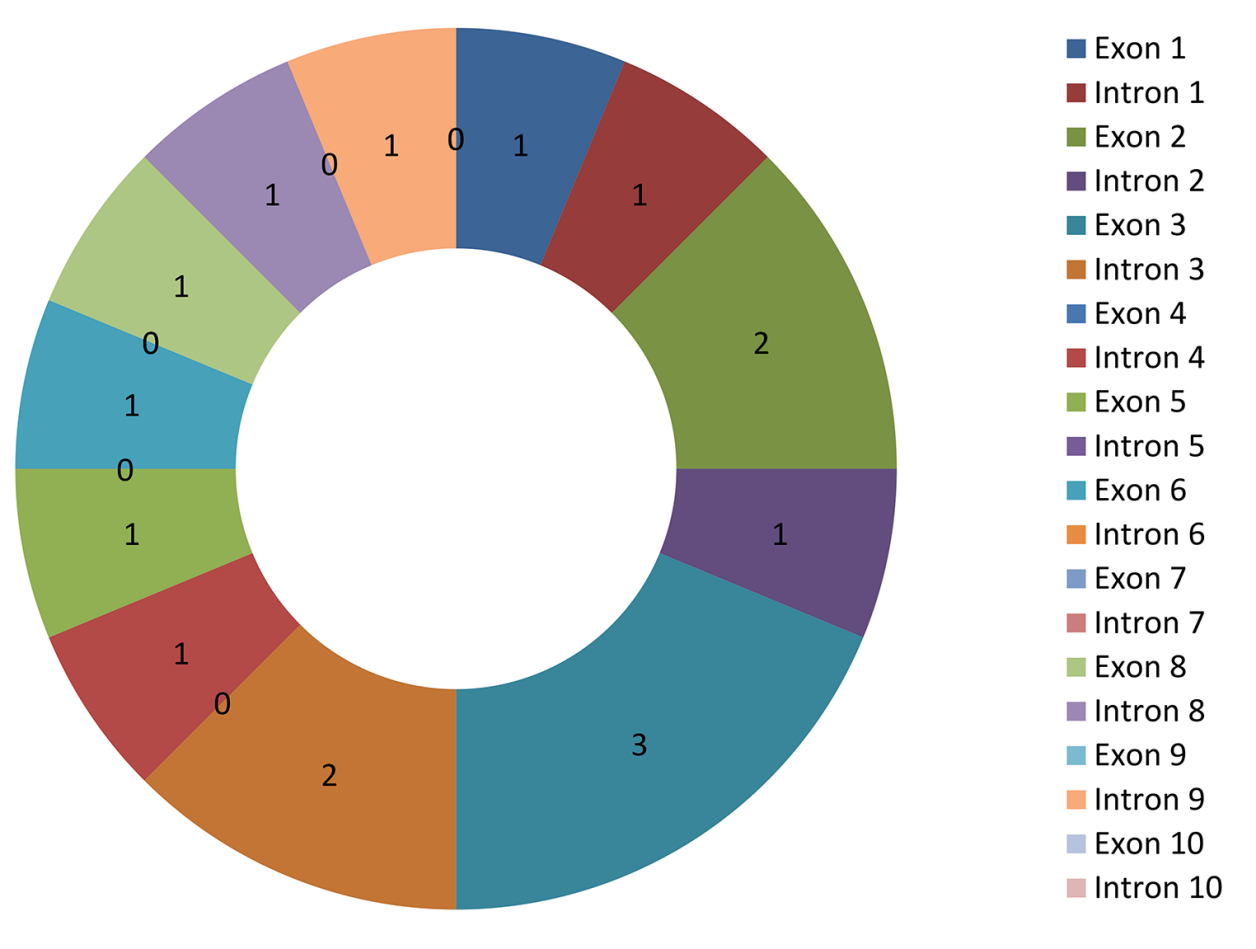
Articles by genomic regions

Article distributions by genomic regions are displayed in Fig. 5. The findings indicated that only 53% (*n* = 8) of the included articles identified the genomic regions while the remaining 47% of the included articles did not indicate the genomic regions. Furthermore, the results showed that two (13%) (Agung et al. 2017; Arango et al. 2014) out of 15 included articles found SNPs in intron 3 and in exon 2 (Zhang et al. 2011; Martinez et al. 2016).

### Identified single nucleotide polymorphisms

The identified and non-identified single nucleotide polymorphisms (SNPs) and their regions are presented in Table 3. The results indicated that out of 15 included articles, seven (47%) of them identified SNPs. Three articles identified AG SNPs (Garrett et al. 2008; Putra et al. 2019; Maskur & Arman 2014).

### Genotypic frequencies

Table 3 indicates the genotypic and allelic frequencies from included articles. The findings showed that out of 15 included articles, 11 (73%) of them identified three genotypes per SNP while 4 (27%) articles (cite) did not identify genotypes per SNP. The results indicated that the genotypic frequencies from the reviewed articles ranged from 0.00 to 102.00.

**Table 3:** Identified single nucleotide polymorphisms

Author	Identified SNPs	Genotypic frequencies
Agung et al. (2017)	1047T > C	AA (0.76); AB (0.22); BB (0.02)
Arango et al. (2014)	-	0.77 (+/+); 0.20 (+/-); 0.03 (-/-)
Beauchemin et al. (2006)	-	0.43 (+/+); 0.42 (+/-); 0.15 (-/-)
China et al. (2007)	-	-
Fathoni et al. (2019)	1180 C > T	CC (0.78); CT (0.21); TT (0.01)
Garrett et al. (2008)	86,273,136 A/G	AA (15.80); AG (51.50); GG (32.70)
Ge et al. (2003)	-	-
Kayumov et al. (2019)	-	VV (0.00); LV (0.37); LL (0.63)
Martinez et al. (2016)	2050 C > G; 2167 C > T; 2200 A > C	-
Putra et al. (2019)	3338 A/G	AA (0.49); AG (0.37); GG (0.14)
Maskur & Arman (2014)	241 A/G	GG (0.09); AG (0.75); AA (0.16)
Ruban et al. (unknown)	-	CC (9.60); CG (48.10); GG; (42.30)
Sedykh et al. (2020)	-	-
Thomas et al. (2007)	-	CC (51.50); CT (34.80); TT (13.70)
Zhang et al. (2011)	4251 C > T	CC (102.00); CT (33.00)

### Allelic frequencies

Identified allelic frequencies from different reviewed articles are presented in Table 4. Out of 15 collected reviewed articles, 13 (87%) of them were clear about allelic frequencies, whereas 2 (13%) were not. The discovered allelic frequencies ranged from the reviewed articles ranged from 0.11 to 94.10.

**Table 4:** Allelic frequencies

Author	Allelic frequencies	
Agung et al. (2017)	A (0.87)	B (0.13)
Arango et al. (2014)	-	-
Beauchemin et al. (2006)	A (5.90)	B (94.10)
China et al. (2007)	-	-
Fathoni et al. (2019)	C (0.89)	T (0.12)
Garrett et al. (2008)	A (41.60)	G (58.40)
Ge et al. (2003)	A (26.90)	B (29.60)
Kayumov et al. (2019)	V (0.18)	L (0.82)
Martinez et al. (2016)	C (67.90)	G (32.10)
Putra et al. (2019)	A (0.67)	G (0.33)
Maskur & Arman (2014)	G (0.47)	A (0.53)
Ruban et al. (unkown)	C (0.34)	G (0.66)
Sedykh et al. (2020)	L (0.69)	V (0.31)
Thomas et al. (2007)	C (68.90)	T (31.10)
Zhang et al. (2011)	C (0.88)	T (0.12)

### Association between identified SNPs’ genotypes and growth traits

The SNPs’ genotypes and their association with growth traits are detailed in Table 5. Out of a total of 15 articles reviewed, 6 articles observed a significant difference (*p* < 0.05) in one or more traits of interest as a result of the SNPs that occur in the GH gene of the cattle. From the 6 articles that were studied 5 traits which included average daily gain (ADG), body weight (BW), weaning weight (WW), yearling weight (YW) and body length (BL), their association with SNPs identified were investigated. About 7 articles (47%) investigated BW with 6 (40%) non-significant and 1 (7%) significantly associated with genotypes. While 7 articles (47%) investigated WW with 5 (33%) non-significant and 2 (13%) significantly associated with genotypes. Three articles (20%) investigated YW with 1 (7%) non-significant and 2 (13%) significantly associated with genotypes. Five articles (33%) investigated ADG with 3 (20%) non-significant and 2 (13%) significantly associated with genotypes. Lastly, one article (7%) investigated BL with significantly associated with genotypes.

**Table 5:** Association between SNP and growth traits

Author	Breed	SNP	Growth traits	Genotypes			Sig
Agung et al. (2017)	Sumba Ongole	1047T > C	BW	AA (25.42)	AB (21.36)	BB (31.70)	ns
			WW	AA (73.24)	AB (77.90)	BB (81.10)	ns
			YW	AA (192.51)	AB (169.20)	BB (123.22)	ns
			ADG	-	-	-	-
			BL	-	-	-	-
			CD	-	-	-	-
Arango et al. (2014)	Holstein	-	BW	-	-	-	-
			WW	-	-	-	-
			YW	-	-	-	-
			ADG	+/+ (0.52)	+/- (0.52)	-/- (0.55)	ns
			BL	-	-	-	-
			CD	-	-	-	-
Beauchemin et al. (2006)			BW	-	-	-	-
			WW	-	-	-	-
			YW	-	-	-	-
			ADG	+/+ (1.51)	+/- (1.49)	-/- (1.47)	*
			BL	-	-	-	-
			CD	-	-	-	-
China et al. (2007)	Charolais	-	BW	AA (22.09)	AB (25.75)	BB (29.57)	*
			WW	AA (129.00)	AB (159.73)	BB (196.86)	*
			YW	-	-	-	-
			ADG	-	-	-	-
			BL	-	-	-	-
			CD	-	-	-	-
Fathoni et al. (2019)	Kebumen Ongole Grade	1180 C > T	BW	CC (29.53)	CT (30.62)	-	-
			WW	CC (127.05)	CT (146.7)	-	-
			YW	CC (297.0)	CT (225.8)	-	-
			ADG	-	-	-	-
			BL	-	-	-	-
			CD	-	-	-	-
Garrett et al. (2008)	Brangus	86,273,136 A/G	BW	AA (36.60)	AG (36.51)	GG (36.84)	ns
			WW	AA (271.85)	AG (267.44)	GG (273.39)	ns
			YW	-	-	-	-
			ADG	AA (1.57)	AG (1.52)	GG (1.51)	ns
			BL	-	-	-	-
			CD	-	-	-	-
Ge et al. (2003)	Angus		BW	-	-	-	-
			WW	-	-	-	-
			YW	-	-	-	-
			ADG	-	-	-	-
			BL	-	-	-	-
			CD	-	-	-	-
Kayumov et al. (2019)	Red Angus		BW	LV (25.5)	VV (24,3)	-	ns
			WW	LV (211.2)	VV (208.9)	-	ns
			YW	LV (303.9)	VV (283.1)	-	*
			ADG	-	-	-	-
			BL	-	-	-	-
			CD	-	-	-	-
Martinez et al. (2016)	BON	-	BW	-	-	-	-
			WW	-	-	-	-
			YW	-	-	-	-
			ADG	-	-	-	-
			BL	-	-	-	-
			CD	-	-	-	
Putra et al. (2019)	Pasundan	3338 A > G	BW	-	-	-	-
			WW	AA (188.35)	AG (199.17)	GG (227.11)	*
			YW	-	-	-	-
			ADG	-	-	-	-
			BL	AA (131.19)	AG (144.31)	GG (108.67)	*
			CD	-	-	-	-
Maskur & Arman (2014)	Bali		BW	GG (15.47)	AG (15.30)	AA (15.24)	ns
			WW	GG (82.14)	AG (80.58)	AA (74.64)	*
			YW	-	-	-	-
			ADG	GG (0.474)	AG (0.417)	AA (0.413)	*
			BL	-	-	-	-
			CD	-	-	-	-
Ruban et al. (unkown)	Angus		BW	-	-	-	-
			WW	-	-	-	-
			YW	-	-	-	-
			ADG	-	-	-	
			BL	-	-	-	-
			CD	-	-	-	
Sedykh et al. (2020)	Hereford		BW	LL (33.4)	LV (32.9)	VV (33.5)	ns
			WW	-	-	-	-
			YW	-	-	-	
			ADG	-	-	-	-
			BL	-	-	-	-
			CD	-	-	-	-
Thomas et al. (2007)	Brangus	-	BW	-	-	-	-
			WW	-	-	-	-
			YW	-	-	-	-
			ADG	AA (1.50)	AG (1.50)	GG (1.60)	ns
			BL	-	-	-	-
			CD	-	-	-	-
Zhang et al. (2011)	Nanyang	4251 C > T	BW	-	-	-	-
			WW	-	-	-	-
			YW	CC (220.12)	CT (229.47)	-	*
			ADG	-	-	-	-
			BL	-	-	-	-
			CD	-	-	-	

BW - Body weight, WW - weaning weight, YW - yearling weight, ADG - average daily gain, BL - body length, CD - chest depth, ns - non-significant, * - significant

## Discussion

Genes involved in controlling the manifestation of growth traits might be interesting as candidate markers for livestock’s performance traits (Ge et al. 2003; Beauchemin et al. 2006). Growth traits are important measurable traits that have a remarkable effect on the beef cattle production industry (China et al. 2007). Understanding the effects of GH gene on growth traits of cattle is important especial when farmers want to develop management strategies focusing on genetic improvement of cattle (Zhang et al. 2011). This systematic review was performed to assess the literature on single nucleotide polymorphisms and their association with growth traits in cattle. A total fifteen (*n* = 15) articles of the fifty-three (*n* = 53) retrieved articles were retained for inclusion and used for development of this systematic review. The findings of this review showed that Angus cattle breed was the most investigated breed (Ge et al. 2003; Kayumov et al. 2019; Ruban et al. unknown) which accounting for 20% of the included articles. This might be a result that the Angus cattle breed is the most dominating popular breed found in the15 countries used on this systematic review. However, in South Africa (SA) the Angus cattle breed is not one of the common breeds reared by communal farmers. Furthermore, the used articles revealed that the 27% of the included articles were from Indonesia followed by 13% of them from Mexico, Russia and United State. This could be due to the availability of resources to perform molecular genetics studies. The results showed that most included articles (87%) used the PCR-RFLP method for genotyping while 13% of the included articles used PCR-SSCP genotyping method (China et al., 2007; Zhang et al., 2011). This could be because the PCR-RFLP genotyping method is among commonly available cheapest genotyping methods in the world (Zhang et al. 2011). Five SNPs (1047T > C, 1180 C > T, 86,273,136 A/G, 3338 A > G and 4251 C > T) were identified from the 15 included articles. The observed results indicated that out of 15 articles used in this systematic review, six (40%) articles showed a significant association between the genotypes of the observed SNPs and growth traits in cattle. Agung et al. (2017) identified 1047T > C SNP and found it to be insignificantly associated with body weight, weaning weight and yearling weight in Sumba Ongole cattle. The SNP 1180 C > T identified by Fathoni et al. (2019) in Kebumen Ongole Grade cattle, showed no significance association with studied traits. The 86,273,136 A/G SNP investigated by Garrett et al. (2008) in Brangus cattle showcased a no significant association with body weight, weaning weight and average daily gain. While Putra et al. (2019) further identified significant correlation between the 3338 A > G SNP and growth traits (weaning weight and body length). On the other hand, Zhang et al. (2011) identified significant correlation between the 4251 C > T SNP and yearling weight in Nanyang cattle. The implication of this systematic review is that the identified SNPs can be used to improve economic important growth traits in cattle breeds. The strength of this systematic review is that to the best of our knowledge there is no similar studies on systematic review of the effects of GH gene on growth traits in cattle. Hence, no studies were used for comparison of the findings. The contribution of this systematic review to the body of knowledge is to suggest that there is a relationship exists between GH gene and some of the growth traits in different cattle breeds. The limitations of this systematic review are (1) some of the articles did not mention the sex of the cattle used which makes it difficult to make the conclusion per sex and (2) no similar SNPs were identified in the included articles to proceed to meta-analysis. This systematic review suggests that there is insufficient evidence on the association between SNPs of GH gene and their association with growth traits in cattle. Therefore, there is a need to conduct more studies that will focus on investigating the relationship between SNPs of GH gene and growth traits in cattle to provide more information and validate these findings to assist on identifying genetic markers that might be used to improve growth traits of cattle during breeding programs.

## Conclusion

In the current study, it was found that the growth hormone gene affects some of the growth traits of cattle and the reported SNPs might be used as possible genetic markers for selection to improve growth traits in cattle during breeding programs. Lastly, the findings of the current study shows that the identified 3338 A > G SNP may be used as a genetic marker for improving weaning weight and body length in during breeding of Pasundan cows.

## Data Availability

All data generated during this study are available through a request to the corresponding author.
